# Novel insights into no-reflow post-PCI: The predictive value of coronary sinus hemodynamics

**DOI:** 10.21542/gcsp.2025.56

**Published:** 2025-10-31

**Authors:** Shimaa Gamal Zein-Elabdeen, Mahmoud Abdelaziz Abdelrashid, Montaser Elsekely, Ahmed Atef Hasan, Mohamed Salah Abdelbasit

**Affiliations:** Cardiology Department, Zagazig University, Faculty of Medicine, Sharkya, Egypt

## Abstract

Background: Coronary no-reflow (NR) represents a significant complication associated with increased mortality in patients with acute myocardial infarction undergoing percutaneous coronary intervention (PCI). The identification of novel predictors for early recognition and timely management warrants investigation in order to improve patient outcomes.

Aim: The coronary sinus has emerged as a versatile tool in various diagnostic and therapeutic interventions. Accordingly, we aimed to measure coronary sinus filling time (CSFT) in patients who developed NR during primary PCI and evaluate its potential role in NR prediction.

Methods: A total of 180 patients with ST-segment elevation myocardial infarction (STEMI) undergoing primary PCI were enrolled in this study. Patients were stratified into two groups based on the occurrence of no-reflow phenomenon: the NR group (*n* = 69) and the normal reflow group (*n* = 111). CSFT was measured following culprit vessel revascularization.

Results: NR developed in 38.3% of patients. CSFT was significantly prolonged in the NR group compared with the normal reflow group (69.13 ± 12.20 vs. 60.19 ± 23.22 frame counts, *p* = 0.001). Regression analysis identified five independent predictors of NR: diabetes mellitus, hypertension, smoking, use of non-compliant balloon, and CSFT. The optimal CSFT cut-off value for predicting NR was >66 frame counts, with a sensitivity of 60.9% and specificity of 78.4%. The area under the receiver operating characteristic curve was 0.722 (*p* = 0.004).

Conclusion: Coronary sinus filling time represents an underutilized yet potentially valuable tool for the prediction and early identification of the no-reflow phenomenon during PCI. Our findings suggest that prolonged CSFT correlates with impaired microvascular perfusion and may serve as an early physiological indicator of impending no-reflow.

## Introduction

No-reflow is a critical phenomenon that may progress with delayed presentation. Angiographic NR following PCI is associated with reduced myocardial salvage, larger infarct size, and increased long-term mortality. Early detection, prevention, and treatment may improve the clinical outcomes of PCI^1^.

NR phenomenon is primarily attributed to microvascular dysfunction, driven by a complex interplay of endothelial swelling, microembolization, capillary plugging, and microvascular spasm, leading to impaired perfusion despite successful epicardial coronary artery recanalization^2^.

Early prediction and the development of novel diagnostic measures are crucial for NR management and myocardial salvage. As CSFT reflects the efficiency of myocardial perfusion, it may serve as a valuable marker for assessing microvascular function. We therefore hypothesized that measuring CSFT could provide insight into the NR phenomenon during primary PCI, making it a potential predictive tool in STEMI patients.

## Methods

### Study design

This prospective cohort study was conducted at the Cardiology Department, Zagazig University Hospital. The Institutional Review Board (IRB) of the Faculty of Medicine reviewed and approved the study protocol (reference number: ZU-IRB#9747/11-10-2022). Written informed consent was obtained from all patients. The study was conducted in accordance with the ethical principles of the World Medical Association Declaration of Helsinki for studies involving human subjects.

Between August 2023 and March 2025, 180 STEMI patients were recruited. Patients were diagnosed based on current European Society of Cardiology guidelines, presented within 24 h of symptom onset, and underwent successful primary PCI. Clinical presentation, electrocardiographic findings, laboratory results, and catheterization laboratory records were collected for all patients. Patients with cardiogenic shock, those who received thrombolytic therapy, and cases with failed PCI were excluded.

Blood flow in the successfully opened infarct-related artery was graded according to the Thrombolysis in Myocardial Infarction (TIMI) flow grading system. NR was diagnosed when angiographic TIMI flow grade was ≤2^4^. CSFT (in frame count) was defined as the interval from the first frame showing contrast at the coronary ostium when the culprit vessel was completely opacified (when contrast dye visibly reached a standardized distal landmark in the artery and filled it fully and consistently across successive frames)^5^ to the frame when contrast reached the coronary sinus (CS) origin.

Coronary angiography was performed at a rate of 15 frames per second. For left coronary arteries, 6–8 mL of contrast was injected at a rate of 2 mL/s using an automated injector system. Vascular access was obtained via either the femoral or radial artery. CSFT was calculated by offline analysis of coronary angiograms and compared between groups. CSFT in seconds was calculated by dividing the frame count by 15. A dedicated semi-automated offline analysis software (ImageJ with time-calibrated DICOM viewer) was used to reduce observer variability. All angiograms were ensured to be of sufficient quality with minimal overlap or foreshortening and were obtained in standard views demonstrating the full course of the vessel.

To ensure measurement reliability, a second independent observer repeated the analysis while blinded to the initial results to assess inter-observer variability and reduce measurement bias.

### Statistical analysis

The chi-squared test was used for comparing categorical variables. Student’s *t*-test or Mann–Whitney U test were used for comparing continuous variables depending on whether they were normally or non-normally distributed, respectively. Statistical significance was defined as *p* < 0.05 with a 95% confidence interval. Cases with missing values were examined; variables with less than 5% missing data were imputed using mean substitution. For multivariate analyses, listwise deletion was employed to maintain consistency. Statistical analyses were performed using IBM SPSS Statistics version 23 software.

## Results

The mean age of the study population was 59.30 ± 10.64 years. Among the cohort, 51 patients (73.9%) were male and 18 (26.1%) were female. NR developed in 38.3% of the study population. Demographic and clinical data are presented in Table 1. Patients in the NR group demonstrated a higher prevalence of hypertension, diabetes mellitus, peripheral arterial disease (PAD), smoking, elevated baseline creatinine levels, and more delayed presentation from symptom onset.

**Table 1:** Demographic and clinical data between groups.

**Variable**	**NR group** **(*N* = 69)**	**Normal flow group** **(*N* = 111)**	**Test of sig.**
**Age (years)**	59.30 ± 10.64	58.68 ± 10.79	^t^ = 0.386^P^ = 0.700
**Males**	51 (73.9%)	78 (70.3%)	^χ^2^^ = 0.278^P^ = 0.598
**Females**	18 (26.1%)	33 (29.7%)	
**Hypertension**	48 (69.6%)	42 (37.8%)	^χ^^2^ = 17.133 ^P^ = < 0.001^*^
**Diabetes mellitus**	54 (78.3%)	36 (32.4%)	^χ^2^^= 35.746 ^P^ = < 0.001^*^
**Dyslipidaemia**	18 (26.1%)	36 (32.4%)	^χ^2^^ = 0.816^P^ = 0.366
**PAD**	6 (8.7%)	0 (0%)	^FET^ = 9.985^P^ = 0.003^*^
**Smoking**	45 (65.2%)	54 (48.6%)	^χ^2^^ = 4.720^P^ = 0.030^*^
**Onset of chest pain (hours)**	5 (1–12)	3 (0–12)	^z^ = −2.500 ^P^ = 0.003^*^
**Admission SBP (mmHg)**	133.48 ± 19.91	132.16 ± 17.50	^t^ = 0.471^P^ = 0.639
**Admission DBP (mmHg)**	83.04 ± 16.08	80 ± 13.12	^t^ = 1.403^P^ = 0.162
**Admission HR (B/min)**	85.61 ± 16.01	84.19 ± 20.88	^t^ = 0.488^P^ = 0.626
**Basal troponin**	366 (10–1200)	200 (10–4500)	^z^ = −0.1018 ^P^ = 0.310
**Basal serum creatinine (mg/dl)**	1.12 ± 0.24	0.98 ± 0.20	^t^ = 4.110 ^P^ = < 0.001^*^
**KILLIP CLASS I**	63 (91.3%)	102 (91.9%)	^FET^ = 0.019^P^ = 1.000
**KILLIP CLASS II**	6 (8.7%)	9 (8.1%)	

**Notes.**

t Independent samples t-test *χ*^2^ Chi-square test z Mann-Whitney U-test FET Fischer’s exact test PAD peripheral artery disease

* Statistically significant (*P*<0.05).

Baseline angiographic characteristics are presented in Table 2. A higher percentage of NR cases occurred with non-compliant (NC) balloon usage (82.6%) compared with the normal flow group (18.9%, *p* < 0.001). The NR group demonstrated significantly prolonged CSFT measured at the culprit vessel (69.13 ± 12.20 frame counts vs. 60.19 ± 23.22 frame counts in the control group, *p* = 0.0001). Similarly, CSFT measured at the non-culprit vessel was more prolonged in the NR group than in the normal flow group (49.48 ± 13.04 vs. 44.86 ± 14.74, *p* = 0.032).

**Table 2:** Angiographic characteristics of patients in both groups.

**Variable**	**NR group (*N* = 69)**	**Normal flow group (*N* = 111)**	**Test of sig.**
**Anterior MI**	39 (56.5%)	54 (48.6%)	^χ^2^^ = 12.758^P^ = 0.002^*^
**Inferior MI**	24 (34.8%)	57 (51.4%)	
**Lateral MI**	6 (8.7%)	0 (0%)	
**Culprit vessels**			
**LAD**	39 (56.5%)	54 (48.6%)	^χ^2^^ = 1.069^P^= 0.586
**RCA**	24 (34.8%)	45 (40.5%)	
**LCX**	6 (8.7%)	12 (10.8%)	
**PTCA**	12 (17.4%)	33 (29.7%)	^FET^ = 3.455*P* = 0.077
**NC ballooning**	57 (82.6%)	21 (18.9%)	^χ^2^^ = 70.289^P^< 0.001^*^
**CSFT of culprit vessel**	69.13 ± 12.20	60.19 ± 23.22	^t^ = 3.413^P^ = 0.001^*^
**Non-culprit vessels**			
**Normal**	39 (56.5%)	69 (56.8%)	^χ^2^^ = 0.001^P^ = 0.975
**Diseased**	30 (43.5%)	48 (43.2%)	
**CSFT of non-culprit vessel**	49.48 ± 13.04	44.86 ± 14.74	^t^ = 2.156^P^ = 0.032^*^

**Notes.**

FET Fischer’s exact test *χ*^2^ Chi-square test NC non compliant

* Statistically significant (*P*<0.05).

**Figure 1.**
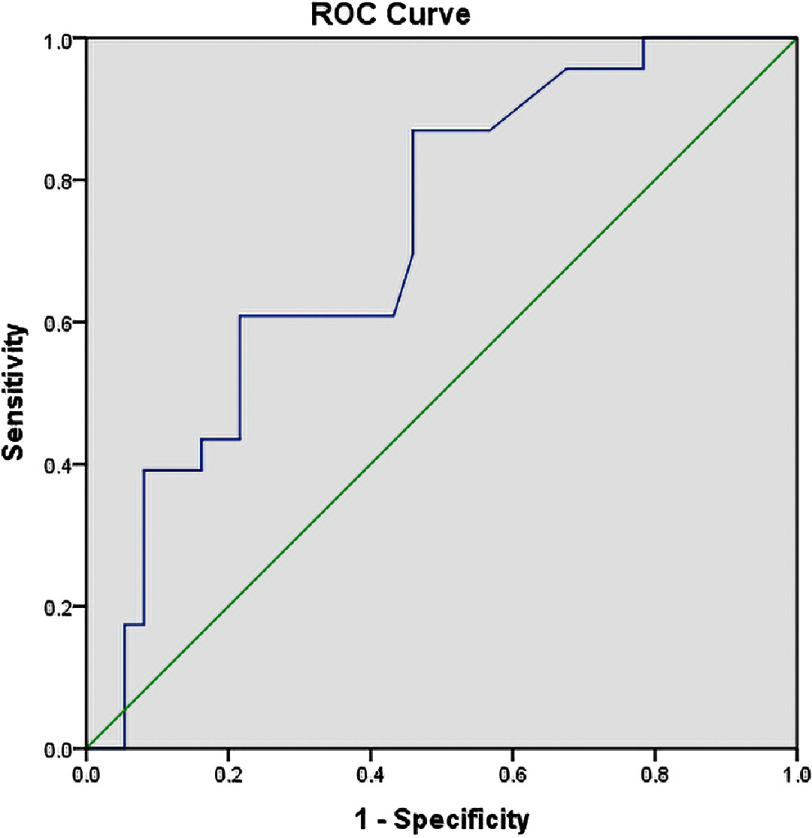
ROC Curve of CSFT of culprit vessel to predict NR phenomena.

Regarding predictors of the NR phenomenon, hypertension, diabetes mellitus, and smoking were identified as clinical predictors, while NC balloon usage and CSFT were identified as angiographic predictors for NR as shown at Table 3. A CSFT cut-off value of >66 frame counts predicted the NR phenomenon during PCI with 78.4% specificity and 72.4% positive predictive value (PPV) Table 4 & Fig. 1.

**Table 3:** Predictors of NR phenomenon obtained from univariate and multivariate logistic regression analysis.

**Variable**	**Unadjusted OR (95% CI)**	**P value**	**Adjusted OR (95% CI)**	**P value**
**CSFT of culprit**	1.024 (1.007–1041)	0.006	1.042 (1.005–1.081)	0.026
**CSFT of non- culprit vessel**	1.024 (1.002–1.046)	0.034	0.921 (0.871–0.973)	0.003
**Diabetes mellitus**	7.5 (3.7–15.1)	<0.001	2.27 (1.98–2.69)	<0.001
**Hypertension**	3.8 ( 2.0–7.1)	<0.001	8.2 (2.3–29.6)	0.001
**Smoking**	2.0 (1.1–3.7)	0.031	6.1 (1.2–30.5)	0.028
Use of NC balloon	20 (9.3–44.5)	<0.001	11.9 (12.03–17.4)	<0.001

**Table 4:** Predictive value of CSFT of culprit vessel (frame count) to NR phenomena.

Diagnostic criteria	CS filling of culprit vessel (time frame rate count)
AUC	0.722
Cut off point	≥66
Sensitivity	60.9%
Specificity	78.4%
NPV	66.3%
PPV	72.4%
Accuracy	68.6%
*P* value	<0.001*

**Notes.**

AUC area under the curve NPV Negative predictive value PPV Positive predictive value P probability

## Discussion

Primary PCI is the reperfusion strategy of choice for patients with STEMI. However, suboptimal myocardial perfusion occurs in a proportion of patients even after successful recanalization of the culprit vessel, a phenomenon known as NR^1^. NR has multiple pathophysiological mechanisms and may complicate 5% to 32% of cases, portending poor short- and long-term prognosis^6^.

Multiple diagnostic modalities target early detection of NR in patients undergoing primary PCI. Coronary angiography is a simple and readily accessible method in the catheterization laboratory. Thrombolysis in Myocardial Infarction (TIMI) flow and corrected TIMI frame count (cTFC) evaluate epicardial blood flow, while TIMI myocardial perfusion grade (TMPG) and myocardial blush grade (MBG) assess microvascular flow^7^.

CSFT is the time required for contrast to traverse the coronary microvasculature and reach the coronary sinus from the injection site. It has been investigated as a marker of coronary microvascular dysfunction in contemporary research^8^. NR phenomenon results from various pathological disturbances at the coronary microvascular level with prolonged blood flow transit time inspired us to investigate the potential role of CSFT in NR prediction. To the best of our knowledge, this is the first study to examine this relationship.

Our prospective study included 180 STEMI patients who underwent primary PCI in the Cardiology Department, Zagazig University Hospitals. Patients were stratified into two groups based on TIMI flow grade following the procedure: Group A (NR group) with TIMI flow grade ≤2, and Group B (normal reflow group) with TIMI flow grade ≥3.

The incidence of angiographic NR during primary PCI varies widely in the literature, ranging from 5% to 32%^1,8^. In our study, NR occurred in 38.3% of the study population, which is consistent with, though moderately higher than, rates reported in multiple studies^9,10^.

We found that the prevalence of hypertension, diabetes mellitus, and PAD was significantly higher among patients with NR [48 (69.6%), 54 (78.3%), and 6 (8.7%), respectively; *p* < 0.001, *p* = 0.001, and *p* = 0.003], reflecting possible underlying microvascular dysfunction predisposed by these conditions. These findings are consistent with those of Zhang et al. and Jonny et al., who also identified these risk factors in NR cases^9,10^.

We also found that delayed presentation and elevated baseline serum creatinine were associated with increased risk of NR. These findings are consistent with previous studies^11,12^.

There was no association between NR occurrence and culprit vessel location (*p* = 0.586), which is consistent with the pathophysiology of microvascular dysfunction. This finding aligns with multiple studies that did not identify any association between infarct location and the risk of NR^10,12^. However, CSFT (in frame counts) showed a significant difference between groups, with greater prolongation in the NR group (69.13 ± 12.20 vs. 60.19 ± 23.22, *p* < 0.001).

Similar CSFT values have been reported in studies assessing microcirculatory function in patients with angina and cardiac syndrome X. Vellani and Haridasan et al. demonstrated that mean CSFT was prolonged in the angina group compared with the normal control group (63.76 ± 10.7 vs. 52.06 ± 5.0, *p* = 0.001)^13^. Similarly, Shakerian et al. showed comparable prolongation in CSFT in the angina group compared with the control group (47.2 ± 9.9 vs. 32.2 ± 3.0, *p* = 0.0001)^3^.

We also measured CSFT in the non-culprit vessel and found significant prolongation in the NR group, which supports the presence of global myocardial microvascular dysfunction that may exacerbate the degree of microvascular obstruction developing after infarct-related PCI.

We also found that utilization of NC balloon to optimize stent expansion was associated with a higher incidence of NR (82.6% vs. 18.9% in the normal reflow group, *p* < 0.001). It is well established that this strategy carries a higher risk of slow flow or NR due to distal embolization. These findings are consistent with those of Parkash et al. ^8^ and Gao et al. ^14^, who reported higher rates of NR with NC balloon use.

Univariate and multivariate regression analyses identified CSFT of the culprit and non-culprit vessels, diabetes mellitus, hypertension, smoking, and NC balloon use as independent predictors of the NR phenomenon during primary PCI. The optimal CSFT cut-off value of ≥66 frame counts predicted the NR phenomenon with 78.4% specificity, 60.9% sensitivity, 72.4% positive predictive value (PPV), and 60.3% negative predictive value (NPV) (*p* = 0.004).

While these traditional predictors (hypertension, diabetes mellitus, and smoking) remain valuable, they are nonspecific and often not actionable in real time. In contrast, CSFT offers immediate feedback during PCI and may help identify microvascular dysfunction even in cases where TIMI flow appears normal, thereby serving as a real-time warning sign to initiate procedural adjustments and protective strategies (e.g., vasodilators, slower balloon inflation, or use of thrombectomy devices).

An integrated approach combining both clinical risk factors, biochemical parameters^15^ and CSFT may offer the most accurate prediction of no-reflow and improve patient outcomes. CSFT provides a functional, dynamic, and potentially actionable complement to static clinical data, with the potential to refine risk stratification.

## Conclusion

No-reflow requires a multifaceted approach for prediction, combining clinical assessment, angiographic findings, and advanced imaging techniques. CSFT serves as a simple, reliable tool for predicting and monitoring NR. Its assessment can provide crucial insights into microvascular integrity, allowing for early identification of high-risk patients and implementation of potential intervention strategies.

### Limitations

This study has several limitations that should be acknowledged.

First, the single-center design may limit the generalizability of the findings. Local institutional protocols, operator experience, and patient demographics at our center may not reflect broader clinical settings, potentially affecting the external validity of the results.

Second, coronary sinus pressure measurements were not performed in our catheterization laboratory, primarily due to the critical clinical situation of patients. However, the absence of direct pressure measurements limits the comprehensive assessment of coronary microvascular resistance, which could have provided further mechanistic insights into the no-reflow phenomenon.

Third, there is a possibility of selection bias in patient inclusion. Despite efforts to apply consistent criteria, unmeasured confounders or referral patterns may have led to a study population with overrepresentation of higher-risk individuals, which may have influenced the observed incidence of no-reflow.

Future multicenter studies with larger, more diverse populations and the incorporation of invasive hemodynamic assessments are warranted to validate and expand upon these findings.

## Funding source

There was no funding for this study.

## Conflicts of interest

All authors report no conflicts of interest.

## Data availability

The data generated or analyzed during our study are not publicly available due to data protection reasons but are available from the corresponding author on reasonable request

## Author contributions

Conceptualization & Design, Analysis and revision: Montaser Elsekelly and M. Abdelbasit

Data Analysis & Interpretation: M. Abdelaziz and A. Atef

Data Analysis, Interpretation & Revision: Shimaa Gamal Zein-Elabdeen

## References

[1] Rezkalla SH, Dharmashankar KC, Abdalrahman IB (2010). No-reflow phenomenon following percutaneous coronary intervention for acute myocardial infarction: incidence, outcome, and effect of pharmacologic therapy. J Interv Cardiol..

[2] Zhao X, Han J, Zhou L (2023). High mobility group box 1 derived mainly from platelet microparti-cles exacerbates microvascular obstruction in no reflow. Thromb Res.

[3] Shakerian Farshad, Panahifar Nasrin (2019). Coronary sinus filling time as a marker of microvascular dysfunction in patients with angina and normal coronaries. Research in Cardiovascular Medicine.

[4] Sabatine MS, Braunwald E (2021). Thrombolysis in myocardial infarction (TIMI) study group: JACC focus seminar 2/8. J Am Coll Cardiol.

[5] Gibson CM, Cannon CP, Daley WL (1996). TIMI frame count: a quantitative method of assessing coronary artery flow. Circulation.

[6] Ndrepepa R, Colleran G, Kastrati A (2018). Noreflow after percutaneous coronary intervention: a correlate of poor outcome in both persistent and transient forms. EuroIntervention.

[7] Rezkalla SH, Kloner RA (2008). Coronary no-reflow phenomenon: from the experimental laboratory to the cardiac catheterization laboratory. Catheter Cardiovasc Interv.

[8] Parkash C, Mal V, Shaikh MA (2023). Clinical characteristics of patents who developed slow flow/ no-reflow after post-dilatation with non-compliant balloon during primary percutaneous coronary intervention. Pak Heart J.

[9] Zhang L, Lin J, Luo L (2024). Analysis of risk factors for PCI no-reflow in coronary heart disease and construction of related prediction models. American Journal of Translational Research.

[10] Fajar JK, Heriansyah T, Rohman MS (2018). The predictors of no reflow phenomenon after percutaneous coronary intervention in patients with ST elevation myocardial infarction: A meta-analysis. Indian Heart Journal.

[11] Annibali G, Scrocca I, Aranzulla TC, Meliga E, Maiellaro F, Musumeci G (2022). “No-reflow” phenomenon: A contemporary review. J Clin Med.

[12] Hala Mahfouz Badran, Ahmed Abdel Fatah, Ghada Soltan (2020). Platelet/lymphocyte ratio for prediction of no-reflow phenomenon in ST-elevation myocardial infarction managed with primary percutaneous coronary intervention. Journal of Clinical and Translational Research.

[13] Haridasan V, Nandan D, Raju D, Rajesh GN, Sajeev CG, Vinayakumar D (2013). Coronary sinus filling time: A novel method to assess microcirculatory function in patients with angina and normal coronaries. Indian Heart J.

[14] Gao P, Lin W, Wang H, Du FH (2018). Application of post-dilation in ST-segment elevation myocardial infract patients undergoing primary percutaneous coronary intervention. Int J Clin Experim Med..

[15] Fedai H, Sariisik G, Toprak K, Taşcanov MB, Efe MM, Arğa Y, Doğanoğulları S, Gez S, Demirbağ R (2024). A machine learning model for the prediction of no-reflow phenomenon in acute myocardial infarction using the CALLY index. Diagnostics (Basel).

